# A Flagellar Glycan-Specific Protein Encoded by *Campylobacter* Phages Inhibits Host Cell Growth

**DOI:** 10.3390/v7122964

**Published:** 2015-12-16

**Authors:** Muhammad Afzal Javed, Jessica C. Sacher, Lieke B. van Alphen, Robert T. Patry, Christine M. Szymanski

**Affiliations:** 1 Alberta Glycomics Centre and Department of Biological Sciences, CW-405 Biological Sciences Building, University of Alberta, Edmonton, AB, T6G 2E9, Canada; majavedlh@yahoo.com (M.A.J.); sacher@ualberta.ca (J.C.S.); lieke.van.alphen@mumc.nl (L.B.A.); rpatry@ualberta.ca (R.T.P.); 2 Saskatoon Research Centre, Agriculture and Agri-Food Canada, 107 Science Place, Saskatoon, SK, S7N 0X2, Canada; 3 Department of Medical Microbiology, Maastricht University Medical Centre, 6202 AZ Maastricht, The Netherlands

**Keywords:** *Campylobacter*, bacteriophage, receptor binding protein, growth inhibition, glycan binding protein, flagellar glycosylation, bacteriostatic

## Abstract

We previously characterized a carbohydrate binding protein, Gp047, derived from lytic *Campylobacter* phage NCTC 12673, as a promising diagnostic tool for the identification of *Campylobacter jejuni* and *Campylobacter coli*. We also demonstrated that this protein binds specifically to acetamidino-modified pseudaminic acid residues on host flagella, but the role of this protein in the phage lifecycle remains unknown. Here, we report that Gp047 is capable of inhibiting *C. jejuni* growth both on solid and liquid media, an activity, which we found to be bacteriostatic. The Gp047 domain responsible for bacterial growth inhibition is localized to the C-terminal quarter of the protein, and this activity is both contact- and dose-dependent. *Gp047* gene homologues are present in all *Campylobacter* phages sequenced to date, and the resulting protein is not part of the phage particle. Therefore, these results suggest that either phages of this pathogen have evolved an effector protein capable of host-specific growth inhibition, or that *Campylobacter* cells have developed a mechanism of regulating their growth upon sensing an impending phage threat.

## 1. Introduction

Bacteriophages (phages) are specific for their hosts, and this specificity is largely directed by their receptor binding proteins (RBPs). Infection of bacteria by lytic phages results in the lysis of host cells, providing an effective means to control bacterial pathogens in the environment and to treat bacterial infections. With the rise in antibiotic resistance among bacterial pathogens, possibilities of exploiting phages for pathogen control are being explored in many research laboratories worldwide. For instance, phages have been shown to reduce *Campylobacter* load from chicken flocks [1,2], and to effectively treat *Mycobacterium ulcerans* infection in a mouse footpad model [3]. Although phage therapy continues to show promise, the use of whole phage particles to treat infections has been hampered due to the possible spread of virulence-associated genes among bacterial pathogens through phage transduction. For this reason and others, the use of phage-derived proteins offers a suitable alternative to whole phage with no risk of genetic exchange between pathogens.

A commonly studied example of a phage-derived RBP that has been tested for its therapeutic potential is the *Salmonella enterica* serovar Typhimurium P22 phage RBP, Gp9, which binds and cleaves the repeating α-d-mannose-(1,4)-α-l-rhamnose-(1,3)-α-d-galactose *O*-antigen of *Salmonella* lipopolysaccharide (LPS) [4,5,6]. We have previously shown that the phage P22 RBP reduces bacterial motility and can be used to effectively reduce *Salmonella* load in chickens [7]. It has also been reported that the phage AF can degrade *Pseudomonas putida* biofilms, an ability conferred by the RBP of the phage [8]. In addition, phage RBPs have been used to effectively target bacterial killing molecules to specific bacterial cells, such as *Escherichia coli* O157:H7 [9] and *Clostridium difficile* [10]. In addition to phage RBPs, other phage proteins such as endolysins, which break down the peptidoglycan of specific hosts, are also being successfully exploited as therapeutics [11,12]. Overall, phage proteins represent a vast reservoir of exploitable proteins for therapeutic and diagnostic applications [13], particularly as they tend to display high affinities for glycan-specific targets. This property makes them excellent candidates for targeting bacteria, which tend to be extensively adorned with carbohydrates (for a recent review on the diversity of glycan-binding proteins encoded by phages, please see Simpson *et al.* [14]).

Previously, in order to explore diagnostic and therapeutic applications against *Campylobacter jejuni* and *Campylobacter coli*, which are major causative agents of foodborne illness worldwide, our group sequenced the genome of a lytic *Campylobacter* phage, NCTC 12673. We identified a putative RBP in its genome, Gp047, based on similarities in size and synteny to RBPs of other characterized phages in the family *Myoviridae* [15]. Recombinant glutathione-*S*-transferase (GST)-fused Gp047 expressed in *E. coli* was found to form sodium dodecyl sulfate (SDS)-resistant multimers, similarly to the P22 phage tailspike protein (TSP), and anti-Gp047 antibodies raised in rabbits were found to cross-react with *Salmonella* phage P22 TSP trimers [15]. However, these antibodies did not bind to the NCTC 12673 phage particle [16], and Gp047 was not detected upon proteomic analysis by mass spectrometry of purified phage virions [15]. Together, these results indicate that Gp047 is not a structural component of the phage particle. Furthermore, although the NCTC 12673 phage has been shown to recognize capsular polysaccharides (CPS) on its target host cells [17], we found that Gp047 recognizes acetamidino-modified pseudaminic acid on host flagella, agglutinating cells and reducing their motility upon binding [16]. Interestingly, Gp047 also shows a broader host recognition range compared to the phage from which it was derived [18]. For these reasons, we have hypothesized that Gp047 is unlikely to be an RBP of this phage, and may instead function as an effector protein in the phage lifecycle (Table 1).

Regardless of its identity, we have shown that Gp047 can be immobilized onto solid surfaces and will specifically capture *C. jejuni* and *C. coli*, two pathogens routinely associated with campylobacteriosis [19]. The Gp047 binding activity is localized in the C-terminal quarter of the protein (CC-Gp047) [18], and this domain can be exploited in assays for the simultaneous detection of these two pathogens [13,18,19] and for the separation/enrichment of campylobacters from complex food samples [20].

Due to the unusual nature of this protein’s glycan binding properties paired with the fact that all *Campylobacter* phages sequenced to date express a homologue of this protein, we speculated that the conserved nature of this protein indicates an important role in the phage lifecycle*.* We hypothesized that Gp047 may function as an extracellular effector that is released upon phage-induced cell lysis where it binds and reduces the motility of nearby cells, thus providing an advantage to newly released phages attempting to attach to their characteristically highly motile hosts. An alternative hypothesis is that Gp047 acts intracellularly during phage infection by binding to flagellar glycans preventing filament assembly, perhaps as a means of diverting resources from energetically costly flagella toward phage replication.

**Table 1:** Summary of Gp047 properties characterized to date.

Property	Reference
Binds to *C. jejuni* and *C. coli* flagella	[16]
Recognizes acetamidino-modified pseudaminic acid residues	[16]
Binding activity localized to C-terminal quarter of the protein	[16]
Agglutinates host bacterial cells	[16,18]
Forms multimers	[15]
Reduces host bacterial motility	[16]
Not identified as a component of the structural phage proteome	[15]
C-terminal homologues encoded by all sequenced *Campylobacter* phages	[21,22]
Effectively captures *C. jejuni* and *C. coli* from complex samples	[20]
Inhibits host bacterial growth	This study
Growth inhibition domain localized to C-terminal quarter	This study
Expression of Gp047 in *C. jejuni* 11168 cells does not affect flagellar phenotype, cell morphology or growth	This study

Here, we report the observation that Gp047 causes bacterial clearance when spotted onto a growing lawn of host cells suspended in agar, and inhibits cell proliferation when added to cells in broth culture. Our observations suggest that zones of observed growth clearance following spotting of phage protein are not necessarily linked to enzymatic degradation of cell surface polysaccharides, as previously reported for other phage proteins, but may instead be the result of bacterial growth inhibition in response to Gp047 binding to their flagella. We also present data suggesting that Gp047 does not affect flagellar assembly when expressed in *C. jejuni* cells. Overall, we have observed that the effect of Gp047 on *C. jejuni* cells appears to involve an extracellular interaction with host cells.

## 2. Materials and Methods

### 2.1. Bacterial Growth Conditions

*C. jejuni* and *C. coli* strains were grown on Mueller Hinton (MH) agar (Difco, Franklin Lakes, NJ, USA) at 37 °C under microaerobic conditions (85% N_2_, 10% CO_2_, and 5% O_2_). *E. coli* strains were grown on LB agar supplemented with ampicillin, kanamycin or chloramphenicol at a final concentration of 100, 50 or 20 µg/mL, respectively, where needed. The list and sources of bacterial strains used in this study are given in Table 2, while plasmids are listed in Table 3.

**Table 2:** List of bacterial strains and mutants used in this study.

Strain or Mutant	Description	Source or Reference
*C. jejuni* 11168	Human isolate	[23]
*C. jejuni* 81-176	Clinical isolate	[24]
11168Δ*kpsM*	Acapsular, *kpsM* mutant in *C. jejuni* 11168	[25]
11168Δ*pseG*	Aflagellate, *pseG* (*cj1312*) mutant in *C. jejuni* NCTC 11168	This study
81-176Δ*pseA*	*pseA* (*cj1316c*) mutant in *C. jejuni* 81-176 (flagellate, no acetamidino modification)	[26]
11168Δ*pseA*	*pseA* mutant in *C. jejuni* NCTC 11168	[16]
11168Δ*pseA:pseA*	11168∆*pseA* mutant chromosomally complemented with wild type *pseA*	[16]
111-28 (pCE111-28/*gp047*)	11168 expressing *gp047* on the pCE 111-28 plasmid	This study
11168 (pCE111-28)	11168 expressing empty pCE 111-28 plasmid	This study

**Table 3:** List of plasmids used in this study.

Plasmid	Description	Source or Reference
pGEX 6P-2	Glutathione-S-transferase (GST) fused protein expression vector, ampicillin resistance marker, *tac* promoter	GE Healthcare
pCE 111-28	*Campylobacter-E. coli* shuttle vector for protein expression, chloramphenicol resistance marker, plasmid pRY111 with σ28 promoter of *flaA*	[27]
pGEX_*gp047*	*gp047* cloned in-frame into multiple cloning site of pGEX 6P-2 for expression of GST-fused Gp047	[15]
pGEX_*ntgp047*	Expression construct of GST-fused N-Gp047 in pGEX 6P-2	[18]
pGEX_*ctgp047*	Expression construct of GST-fused C-Gp047 in pGEX 6P-2	[18]
pGEX_*ccgp047*	Expression construct of GST-fused CC-Gp047 in pGEX 6P-2	[18]
pGEX_*ncgp047*	Expression construct of GST-fused NC-Gp047 in pGEX 6P-2	[18]
pCE_*gp047*	Expression construct of untagged Gp047 for expression in pCE 111-28 in *C. jejuni*	This study

### 2.2. Construction of a PseG Mutant in C. jejuni 11168

A *pseG* mutation in 11168 was generated by transferring this mutation from *C. jejuni* 81-176, where the *pseG* gene was inactivated by transposon insertion [28]. The DNA fragment carrying the *pseG* mutation was amplified from 81-176∆*pseG* by PCR with primers CS1031 (5′-CTACAACATCAAAATTTTTAGCAATATATTC-3′) and CS1032 (5′-CTTTAACTATAGGTGGCGGGATAA-3′) using Platinum^®^ Taq high fidelity DNA polymerase (Invitrogen, Carlsbad, CA, USA). PCR products were purified using the MinElute^®^ PCR purification kit (Qiagen, Hilden, Germany) according to the manufacturer’s protocol. *C. jejuni* 11168 cells were transformed with the PCR product by natural transformation and colonies were selected on MH agar supplemented with chloramphenicol (15 µg per mL). Mutant colonies were confirmed by PCR.

### 2.3. Expression of Gp047 and Its Derivatives

DNA fragments encoding for Gp047 and its truncated versions were cloned into the pGEX 6P-2 vector, expressed in *E. coli* BL21 as GST-fused proteins, and purified as described previously [15,16,18], with the exception that where indicated, Gp047 was used in the GST-column’s elution buffer (10 mM reduced l-glutathione, 50 mM Tris, 1 mM DTT at pH 9.0) without first dialyzing into PBS.

For expression in *C. jejuni* cells, the *gp047* gene was amplified from the phage NCTC 12673 genome using Vent polymerase (New England Biolabs, Ipswich, MA, USA) with primers (5′-ATCTCGAGAAGAAGGAGTGTTATGATAGAACCAAAAAGAGAACCTACACAAG-3′) and (5′-ATGGTACCTTAATTTATATTGAACGCATATATATAAGAACTATCGTTTGTTTC-3′) containing a *Xho*I or a *Kpn*I restriction site (underlined), respectively. The PCR product was purified and ligated into *Xho*I/*Kpn*I-digested pCE 111-28. *C. jejuni* 11168 cells were transformed with the resultant construct by natural transformation, and colonies were selected on MH agar supplemented with chloramphenicol (15 µg per mL). Colonies containing the pCE 111-28/*gp047* construct were confirmed by restriction digest of purified plasmid DNA and by sequencing.

### 2.4. Agar Plate Growth Clearance Assay

Clearance of bacterial growth was tested by spotting phage protein onto a freshly inoculated bacterial suspension using a standard overlay agar method as described for phage plaque assays. Overnight bacterial growth was harvested in brain heart infusion broth supplemented with 1 mM CaCl_2_ and 10 mM MgSO_4_ (final concentration) and set to an OD_600_ of 0.35. A 3-mL aliquot of this suspension was transferred to a 35 × 10 mm polystyrene dish (Corning Inc., Corning, New York, NY, USA) and incubated at 37 °C for 4 h under microaerobic conditions. Then, 100 µL of this suspension was mixed with 5 mL sterile 0.6% molten NZCYM agar (Difco, Franklin Lakes, NJ, USA) cooled to 45 °C. This suspension was poured onto the surface of a previously solidified, pre-warmed NZCYM or MH agar plate containing 1.5% agar. Plates were allowed to solidify for 15 min and then 5–10 µL phage suspension (10^8^ PFU/mL) or sterile protein solution (0.91–1.3 mg/mL) of Gp047, its derivatives or Gp069, a hypothetical protein from phage NCTC 12673 (dissolved in PBS or 10 mM reduced l-glutathione, 50 mM Tris, 1 mM DTT, pH 9.0) was spotted onto the agar surface and allowed to completely soak into the agar. Culture plates were incubated at 37 °C under microaerobic conditions and zones of growth clearance were observed after 18–24 h.

### 2.5. Scanning Electron Microscopy

Spot assays were performed as described above by spotting 5 µL fresh full-length Gp047 in PBS at 0.91 mg/mL. Following overnight growth of lawns, agar squares were excised using a sterile scalpel from either inside, outside or at the interface of the clearance zone. Slabs were then trimmed to leave the top layer (approximately 2 mm) intact and incubated in scanning electron microscopy (SEM) fixative (2.5% glutaraldehyde; 2% paraformaldehyde in 0.1 M phosphate buffer) overnight at 4 °C. Slabs were then prepared for microscopy using methods described by Bozzola and Russell [29]. Briefly, slabs were washed three times in 0.1 M phosphate buffer for 10 min each and then dehydrated by incubating 15 min each in a series of alternating ethanol and hexamethyldisialazane (HMDS) washes. The washes were done as follows: 50% ethanol, 70% ethanol, 90% ethanol, 100% ethanol, ethanol:HMDS 75:25, ethanol:HMDS 50:50, ethanol:HMDS 25:75 and 100% HMDS. Slabs were incubated in just enough HMDS to cover the slab surface, which was left to evaporate overnight. Once fully dried, slabs were mounted onto SEM stubs, sputter-coated with gold using the Hummer sputtering system (Anatech Ltd., Battle Creek, MI, USA) and imaged using the Philips/FEI (XL30) scanning electron microscope (Philips/FEI, Hillsboro, OR, USA) with an electron beam energy of 20 kV.

### 2.6. Liquid Growth Assays

Cells were harvested from overnight MH plate cultures and set to an OD_600_ of 0.03 (approximately 10^8^ colony forming units (CFU)/mL) in MH broth. To each well of a 96-well Nunclon flat bottom plate, 275 µL of this suspension and 25 µL of either Gp047 (0.91 mg/mL) or buffer (10 mM reduced l-glutathione, 50 mM Tris-HCl, 1 mM DTT, pH 9.0) was added. Cultures were set up in triplicate, and OD_600_ measurements were taken using a plate reader at 0, 0.75, 4, 9, 13, 24 and 30 h. Colony counts were done by serially diluting 10 µL aliquots of cell suspension into MH, plating onto pre-dried MH plates and incubating 48 h until single colonies were observed.

### 2.7. Western Blotting

In order to detect Gp047 protein expression in *C. jejuni* 11168, cells transformed with pCE 111-28/*gp047* were grown on MH agar containing 15 µg/mL chloramphenicol. Whole cell lysates were then examined by Western blotting using Gp047-specific polyclonal antisera. Growth from 1 plate per strain was harvested in PBS, frozen, thawed in the presence of 1× Protease Inhibitor Cocktail (Roche, Basel, Switzerland), and sonicated 4 × 30 s. Supernatants were collected, boiled 10 min in the presence of SDS loading buffer and run on a 12% SDS-polyacrylamide gel electrophoresis (PAGE) gel (120 V, 1.5 h). Gels were transferred to polyvinylidene fluoride (PVDF) (30 V, room temperature, 14 h), blocked with 5% skim milk/PBST 1 h, probed with polyclonal anti-Gp047 antibodies (1:4000) 1 h, washed 3 × 5 min in PBST, probed with goat anti-rabbit-AP antibodies (1:10,000) for 1 h, and developed with nitro-blue tetrazolium chloride (NBT)/ 5-bromo-4-chloro-3'-indolylphosphate (BCIP) until bands were visible.

### 2.8. Immunogold Labeling and Transmission Electron Microscopy

*C. jejuni* cells were harvested from a plate of overnight growth in MH broth and set to an OD_600_ of 0.5. Immunogold labeling and transmission electron microscopy was done as described previously [16] with some exceptions. Briefly, Formvar-coated copper grids were floated on drops of the bacterial cell suspensions for 1 h. The grids were washed 3 × 5 min each with blocking solution (PBS containing 5% bovine serum albumin and 0.05% Tween), then blocked with the same solution for 1 h at room temperature (RT) or overnight at 4 °C. The grids were then floated on drops of freshly purified full-length Gp047 (0.91 mg/mL diluted 1:10 in blocking solution) for 1 h at RT or overnight at 4 °C. The grids were washed as above, then floated on rabbit anti-Gp047 antibody solution [15] (diluted 1:200 in blocking solution) for 1 h at RT. After washing again as above, the grids were then floated on goat anti-rabbit IgG (whole molecule) conjugated to 10-nm gold particles (BB International), diluted 1:200 in blocking solution, for 1 h at RT. The grids were then washed 3 × 5 min with blocking solution, 5× with PBS, 5× with distilled water and then air-dried on Whatman filter paper. The grids were examined using a transmission electron microscope (Philips Morgagni 268; FEI Company, Hillsboro, OR, USA). Images were captured with a charge-coupled device camera and controller (Gatan, Pleasanton, CA, USA) and processed using DigitalMicrograph (Gatan, Pleasanton, CA, USA).

## 3. Results

### 3.1. Gp047 Causes Capsular Polysaccharide-Independent Clearance of C. jejuni Growth

**Figure 1:**
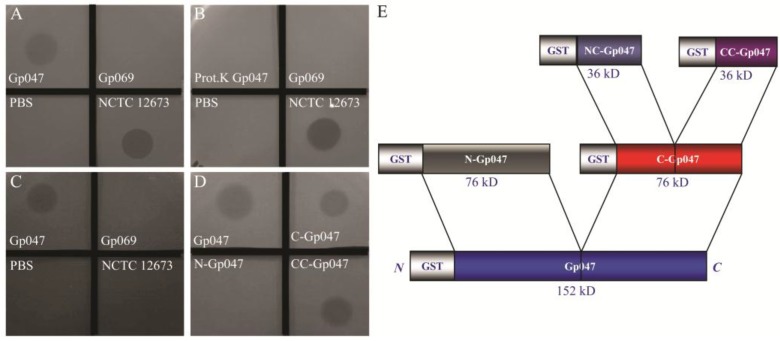
*C. jejuni* growth clearance assays. GST-Gp047 and phage NCTC 12673 showed growth clearance of *C. jejuni* 11168 wild type when spotted on the lawn of growth, while PBS and GST-Gp069 did not show this phenotype (**A**); Growth clearance activity of Gp047 was abolished when the protein was pre-treated with proteinase K before spotting onto a lawn of wild type 11168 growth (**B**); Gp047 was able to cause growth clearance of the CPS mutant 11168Δ*kpsM*, while phage NCTC 12673 was not able to cause lysis of the mutant (**C**); Similar to the full-length Gp047, truncated derivatives C-Gp047 and CC-Gp047 also caused growth clearance of wild type 11168 cells, while N-Gp047 did not (**D**); A schematic of Gp047 derivatives expressed as GST-fused proteins in pGEX-6P2 as described previously [18] is shown for reference (**E**).

When spotted on a growing lawn of *C. jejuni* 11168 in semi-solid medium, recombinant Gp047 caused a hazy zone of growth clearance similar to, yet less complete than, clearing caused by the whole phage NCTC 12673 from which Gp047 was derived (Figure 1). Another protein of unknown function (Gp069) originating from the same phage and purified in the same manner using a GST-tag does not cause a zone of clearance, suggesting that Gp047 is the causative agent of *C. jejuni* growth clearance (Figure 1A). To further confirm this Gp047-related phenotype, the protein was treated with proteinase K before spotting onto the lawn of growth, which resulted in the absence of a zone of clearance (Figure 1B). Previous reports have suggested that incomplete or hazy bacterial growth clearance observed as a halo around some phage plaques is caused by polysaccharide hydrolysis by phage proteins [8]. It has been shown that NCTC 12673 phage recognizes *C. jejuni* CPS [17], so we tested a CPS mutant, 11168Δ*kpsM,* for susceptibility to Gp047-induced growth clearance. As expected, whole NCTC 12673 phage did not form plaques, however, Gp047 was able to cause a zone of clearance on 11168Δ*kpsM* (Figure 1C). These results demonstrate that the growth clearing activity by Gp047 is not a result of CPS hydrolysis as it occurs independently of the presence of CPS.

### 3.2. Growth Clearance-Associated Domain is Localized in the C-Terminal Quarter of Gp047

We previously expressed different lengths of recombinant Gp047 fused with GST (schematic diagram shown in Figure 1E), and found that the binding domain of Gp047 is localized in the C-terminal quarter of the protein (CC-Gp047) [18]. Here, we tested these different truncations of Gp047 for their ability to cause *C. jejuni* growth clearance, and found that the growth clearance-associated domain is also localized in the C-terminal quarter, CC-Gp047 (Figure 1D).

### 3.3. Clearance Phenotype of Gp047 Is Contact- and Dose-Dependent

**Figure 2:**
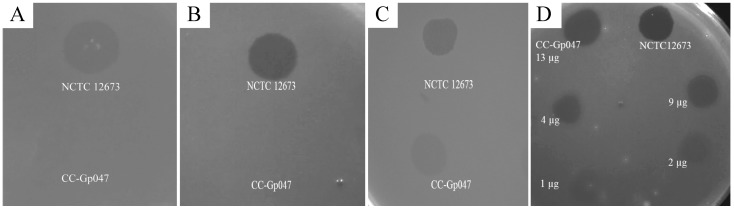
CC-Gp047 did not clear growth of the aflagellate mutant 11168Δ*pseG* (**A**); Growth clearance was also not observed when CC-Gp047 was spotted onto the acetamidino-mutant 11168Δ*pseA* (**B**); Growth clearance was restored when 11168Δ*pseA* was complemented with a wild type copy of the *pseA* gene (**C**); (**D**) CC-Gp047 showed a dose dependent growth clearance when different concentrations (13, 9, 4, 2 and 1 µg) were spotted onto a growing *C. jejuni* lawn.

We previously performed protein affinity chromatography and different binding assays on several *C. jejuni* mutants and found that Gp047 binds to bacterial host flagella through recognition of acetamidino-modified pseudaminic acid residues on flagellin subunits [16]. To test whether binding of Gp047 to bacterial flagella was important for growth clearance activity of this protein, we tested the clearance activity of CC-Gp047 on a lawn of *C. jejuni* 11168Δ*pseG.* This strain has a mutation in an essential enzyme of the pseudaminic acid biosynthesis pathway, which is required for proper flagellar assembly. This mutation therefore results in aflagellate and non-motile bacterial cells [28]. CC-Gp047 did not show growth clearance of 11168Δ*pseG* (Figure 2A).

To verify whether motility or the presence of the flagellar filament was important for the growth clearance activity, we tested 11168Δ*pseA,* a motile strain with flagella displaying pseudaminic acid without the acetamidino modification required for Gp047 binding [16,28]. Here we found that growth of 11168Δ*pseA* was not cleared by CC-Gp047 (Figure 2B). Since decoration of *C. jejuni* flagellar filaments with acetamidino-modified pseudaminic acid is required for Gp047 binding, loss of growth clearance by CC-Gp047 in *C. jejuni* 11168Δ*pseA* suggests that growth clearance by Gp047 is contact-dependent. Complementation of 11168Δ*pseA* with a wild type copy of the *pseA* gene [16] restored growth clearance by Gp047 (Figure 2C), confirming the presence of the Gp047 receptor on bacterial flagella to be crucial for the growth clearance activity of the protein.

To find out whether the clearance potential of Gp047 was dose dependent, we spotted varying concentrations of CC-Gp047 onto the *C. jejuni* plates. The zones of clearance showed increasing intensity as the concentration of CC-Gp047 was increased (Figure 2D), indicating a dose-dependent response.

### 3.4. Zones of Clearance Observed Using Electron Microscopy Show an Altered Growth Pattern

**Figure 3:**
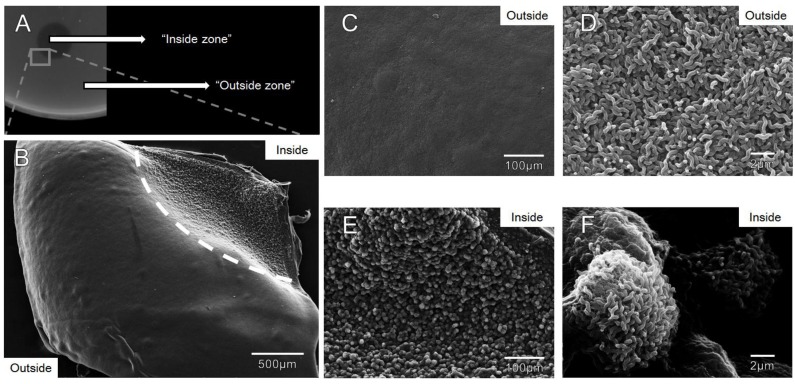
Scanning electron micrographs depicting *C. jejuni* 11168 growth in molten NZCYM agar at the interface where purified full-length Gp047 (0.9 mg/mL) was spotted. (**A**) An image of an excised agar slab where Gp047 was spotted on *C. jejuni* 11168 and the resulting clearance effect observed after 24 h (the grey box shows where the agar slab was excised); (**B**) A low magnification image highlighting the difference in topology between areas exposed to Gp047 (upper right corner) and those that were not exposed (remainder of the square), separated by a dotted white line; (**C**, **D**) Increasingly magnified images of bacterial growth outside the Gp047 spot showing a uniform lawn of *C. jejuni*; (**E**, **F**) Increasingly magnified images of the Gp047-exposed region of the agar plate showing spherical ultrastructures of *C. jejuni*. Images are representative of four replicate experiments.

In order to visualize growth architecture of campylobacters growing within the observed zones of clearance on agar plates, we developed a method to visualize the cells by directly analyzing excised agar slabs by SEM. Agar slabs taken from the interface between clearance zones and outside growth showed a sharp contrast at the interface with a noticeable drop in cell density where protein was spotted (Figure 3A,B). We observed a smooth, uniform lawn in the absence of Gp047 (Figure 3C), but in the presence of the protein, *C. jejuni* cells were grouped in spherical ultrastructures visible even at low magnification (Figure 3E). Individual cell morphology appeared to be unchanged inside and outside the Gp047 zone (Figure 3D,F). For consistency, the images in Figure 3B–F were taken from the same agar slab, however this effect was observed in four replicate experiments. These results provide evidence that Gp047 is bacteriostatic, not lytic, since the cells remaining following treatment were normal in morphology, and no presence of lysed cells or debris was observed.

### 3.5. Gp047 Addition to Broth Cultures Inhibits Cell Growth

**Figure 4:**
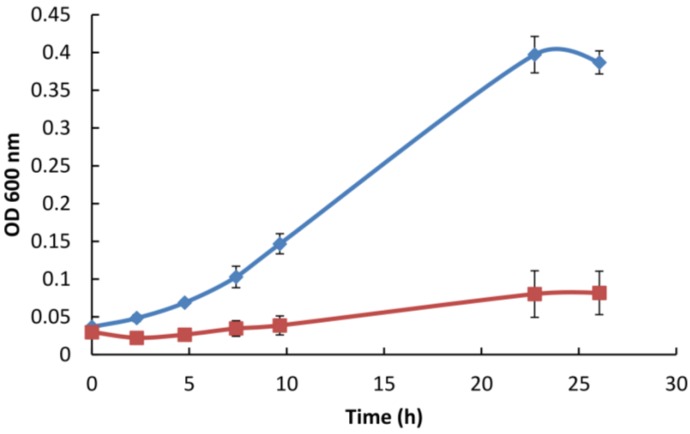
Full-length Gp047 added exogenously to *C. jejuni* 11168 broth cultures inhibits cell growth. GST-Gp047 (75 µg/mL final concentration) was added to cells suspended in MH broth and grown at 37 °C under microaerobic conditions. The blue line represents *C. jejuni* growth with glutathione elution buffer added in place of Gp047, while the red line represents *C. jejuni* growth in the presence of Gp047. Error bars represent the standard error calculated from three technical replicates.

To verify whether the observed Gp047-induced clearing of *C. jejuni* lawns on agar plates was a result of growth inhibition, we added GST-purified full-length (FL)-Gp047 to MH broth inoculated with approximately 10^8^ CFU/mL of *C. jejuni* and proceeded to measure growth by OD_600_ and by plate counting over the course of 26 h. We found that in comparison to the buffer control, the presence of Gp047 resulted in cell growth inhibition as indicated by OD_600_ measurements and plate counts (Figure 4). At *t* = 23 h, control cultures (glutathione elution buffer added in place of Gp047) had grown from an initial concentration of (3.71 ± 0.03) × 10^8^ CFU/mL to (1.96 ± 0.42) × 10^9^ CFU/mL. In contrast, the cell concentration in Gp047-containing cultures remained nearly constant over this time frame, going from (1.22 ± 0.03) × 10^8^ CFU/mL at *t* = 0 to (1.50 ± 0.23) × 10^8^ CFU/mL at *t* = 23 h. These results suggest that cells are not being killed or lysed by the presence of Gp047, but that the protein inhibits their growth resulting in a bacteriostatic effect.

### 3.6. Gp047 Expressed in C. jejuni Cells Does Not Affect the Flagellar Phenotype

**Figure 5:**
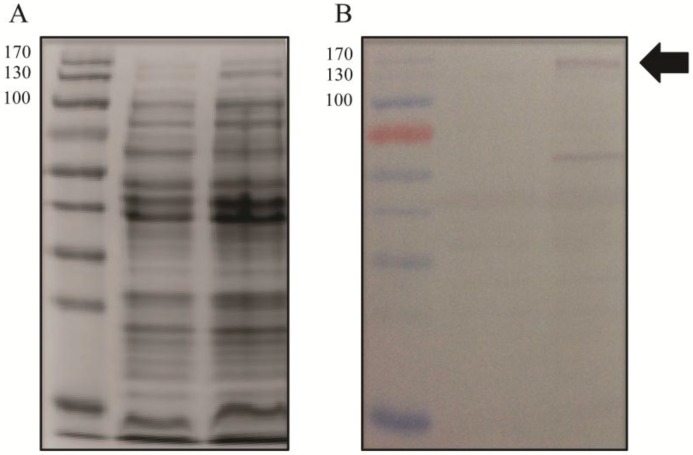
Gp047 can be expressed in *C. jejuni* 11168 cells. Gp047 was expressed in 11168 cells on the pCE 111-28 plasmid and cell lysates of wild type 11168 cells (lanes 1, 3) and 11168/pCE_*gp047* (lanes 2, 4) were subjected to SDS-PAGE stained with Coomassie; (**A**) or transferred to a Western blot and detected with anti-Gp047 antibodies; (**B**) The arrow indicates the expected size for full-length Gp047 (152 kDa). The molecular masses are shown on the left of each panel in kDa.

**Figure 6:**
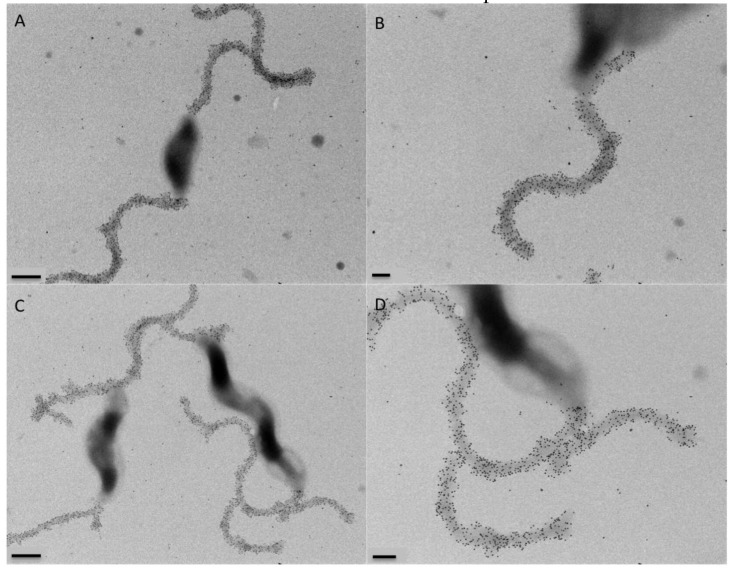
Internal Gp047 expression did not influence binding of externally added Gp047 to *C. jejuni* cells. Gp047-expressing (**C**,**D**) and empty pCE 111-28-expressing (**A**,**B**) *C. jejuni* cells display comparable levels of binding by exogenous Gp047, as shown by anti-Gp047 immunogold labeling. Scale bars represent 500 nm in (**A**,**C**) and 200 nm in (**B**,**D**).

To test the hypothesis that the role of Gp047 in the phage lifecycle is to disrupt the *C. jejuni* flagellar assembly process through sequestration of flagellar glycans within the cytoplasm, we cloned full-length *gp047* and transformed it into *C. jejuni* 11168 cells. Western blotting confirmed that Gp047 was expressed in the resultant cells (Figure 5). However, upon analysis by immunogold labeling of Gp047 binding to its flagellar glycan receptor, we found no difference between flagellar labeling of Gp047-expressing cells and the corresponding empty vector control (Figure 6). We also did not observe any change in flagellar or cell morphology as a result of endogenous expression of Gp047 (Figure 6), nor did the Gp047-expressing strain grow differently than the empty vector control (results not shown).

## 4. Discussion

We recently demonstrated that the *C. jejuni* phage NCTC 12673 binds specifically to acetamidino-modified pseudaminic acid residues displayed on the flagella of most *C. jejuni* and *C. coli* isolates [16]. Furthermore, we have shown that homologues of this protein exist in all sequenced campylobacter phages and that homologues that we tested are also capable of binding to the same sugar residues [16]. These observations were notable considering that many *C. jejuni* phages, including NCTC 12673, recognize the phase-variable capsular polysaccharides and exhibit limited host ranges with high rates of resistance development [17,30]. In addition, it is well known that the 9-carbon sugar known as pseudaminic acid is essential for flagellar filament assembly and thus for bacterial motility and virulence. These observations led us to believe that Gp047 plays an important auxiliary role in the phage lifecycle as opposed to representing an RBP, as was previously thought [15,16].

Interestingly, we found that this protein caused a zone of clearance on agar plates containing *C. jejuni*, which at first glance appeared similar to published reports of surface polysaccharide depolymerization observed previously for other phage proteins. This type of clearing, which can present as a “halo” around phage plaques or as a zone of incomplete clearing on susceptible cell lawns, was directly demonstrated in one case to be caused by Gp19, an exopolysaccharide-degrading RBP from *P. putida* phage AF [8]. However, while we have shown here that Gp047-mediated growth clearance is contact- and dose-dependent, depending on the same C-terminal quarter that is essential for flagellar binding, the fact that the receptor for Gp047 is a sugar monosaccharide on a glycoprotein, paired with our observation that Gp047 clears acapsular mutant cells, suggests a polysaccharide depolymerization-independent mechanism of action.

We tested the possibility that Gp047 could have an intracellular role in *C. jejuni* during phage infection. However, *C. jejuni* cells constitutively expressing Gp047 did not display an altered flagellar morphology or show an obvious difference in the levels of acetamidino-modified pseudaminic acid displayed on their filaments, suggesting that this protein solely has an extracellular role during the phage lifecycle.

We therefore employed a novel SEM-based method to directly observe *C. jejuni* cells on agar at the interface between the Gp047-induced zones of clearance in order to better analyze the effect of this protein on bacterial growth architecture. By excising agar slabs containing cells exposed to Gp047, we were able to see that cells were still present in the clearance zone, but that the smooth, thick layers of cells observed in the absence of treatment appeared disrupted in the presence of the protein. Instead, the remaining cells displayed an altered topology, composed of large, spherical groups of cells surrounded by deep gouges of space in between. We speculate that the first cells to come into direct contact with Gp047 would likely adsorb (and sequester) the protein upon its binding to the flagella (see Figure 6 for an illustration of the abundant binding capacity of Gp047 to a single flagellum, as evidenced by the presence of numerous gold particles along the length of each filament). From this, we hypothesize that Gp047 might be inhibiting the growth of cells it is able to access as it descends into the bacterial layers, but that many cells would be protected from this activity and may still be able to grow and divide within discrete Gp047-free microenvironments. While this is one possible explanation for the altered phenotype observed by SEM as a result of Gp047 application, further research is required to unambiguously determine how Gp047 is causing this phenotype.

We have also observed that Gp047 is capable of inhibiting *C. jejuni* growth in broth culture and that cell counts do not decrease as a result of Gp047 addition to cultures but rather remain constant over a 26-h period. These data suggest that Gp047 is not a bactericidal protein but instead may prevent cell division. Although it seems counterintuitive that a phage would encode a protein with the capacity to inhibit the growth of its own host population, we speculate that by keeping the local population of susceptible targets low, the phage population would benefit by being able to diffuse further from the site of cell lysis before attaching to a susceptible host [31,32]. Since only one phage can productively infect and lyse a given cell, extra phage genomes are wasted when multiple phages irreversibly bind one cell [33]. Therefore, reducing host growth in the local environment might allow a greater number of new hosts to be infected per phage. 

Motility of *C. jejuni* 11168 is also reduced in semisolid media supplemented with Gp047 [16]. It is possible that binding of Gp047 to the bacterial flagellum interferes with flagellar rotation, consequently reducing motility. Unlike the bacterial host, phages are not motile and must reach their hosts by diffusion. Thus, phages could take advantage of the reduced motility of their host to enhance infectivity. Alternatively, Gp047 binding to bacterial flagella might be triggering a mechanism to reduce bacterial metabolic activity and cell division to conserve energy for phage replication.

From another perspective, the growth reduction effect we observe could result from *Campylobacter*-induced growth suppression in response to sensing bound proteins. This type of response could have evolved as a phage defense mechanism [16]. Since some *Campylobacter* phages are flagellotropic [30], campylobacters with this activity might benefit from down-regulating their metabolism in response to their flagella being bound by phages and/or their proteins. In this model, which would be reminiscent of phage restriction or abortive infection [33], cells would reduce their own growth and would reduce the efficiency of phage infection in these cells, giving rise to fewer infectious virions and thus tempering the effect of phages on the overall population. Alternatively, if grown in biofilm or semisolid conditions, exemplified by our agar plates, these sacrificed cells might physically protect other cells lying deeper within the matrix.

Other reports have documented cases of proteinaceous compounds causing cellular growth inhibition, killing or signaling, lending support for this model. Firstly, antibodies represent a well-known class of proteins with bacterial-binding activities that have been shown to affect cell growth by mechanisms other than opsonization and complement-mediated killing, such as through inducing oxidative stress in bound pathogens [34,35]. Additionally, antibodies against *Cryptococcus neoformans* polysaccharides were reported to mediate protection against this pathogen by altering expression of genes associated with fatty acid metabolism and protein translation upon binding [36]. Another study showed that monoclonal antibodies raised against the capsule of this pathogen were able to bind and cause stiffness in the fungal capsule, which, in turn, inhibited cell division by means of impaired budding of the yeast cells [37]. *Streptococcus pneumoniae* viability has also been shown to be impaired as a result of binding by non-opsonic antibodies raised against its CPS. These antibodies were shown to agglutinate bacterial cells, which eventually resulted in increased competence and genetic exchange among the cells, which lead to impaired cell viability [38]. Complement-mediated killing was ruled out in this system.

Interestingly, a recent study has shown that biosynthesis of *C. jejuni* polar flagella in association with the FlhG protein helps regulate cell division in *C. jejuni* [39]*.* These authors suggest that biosynthesis of polar flagella is involved in symmetric cell division, and thus in production of viable daughter cells. We have previously shown that binding of Gp047 causes thickening of bacterial flagella [16] (also shown in Figure 6), and therefore the binding of Gp047 along the length of *Campylobacter* flagella may impair cell division through cellular sensing of altered, perhaps stiffened flagella resulting in a transmission of signals to the cell division machinery.

At this point, it is not clear whether *C. jejuni* cells are able to slow their own growth and/or cell division in response to detecting the presence of proteins bound to their flagella, whether this phage effector protein has its own mechanism of bacteriostatic activity, or whether this interaction between Gp047 and *C. jejuni* exists outside the laboratory environment. We are actively pursuing these questions. Overall, these results point toward novel interactions between *Campylobacter* cells and their phages and may identify a new class of phage effector proteins influencing phage propagation.
